# A laser-microfabricated electrohydrodynamic thruster for centimeter-scale aerial robots

**DOI:** 10.1371/journal.pone.0231362

**Published:** 2020-04-29

**Authors:** Hari Krishna Hari Prasad, Ravi Sankar Vaddi, Yogesh M. Chukewad, Elma Dedic, Igor Novosselov, Sawyer B. Fuller

**Affiliations:** 1 Autonomous Insect Robotics (AIR) Lab, Department of Mechanical Engineering, University of Washington, Seattle, WA, United States of America; 2 Novosselov Research Group, Department of Mechanical Engineering, University of Washington, Seattle, WA, United States of America; Boston University, UNITED STATES

## Abstract

To date, insect scale robots capable of controlled flight have used flapping-wings for generating lift, but this requires a complex and failure-prone mechanism. A simpler alternative is electrohydrodynamic (EHD) thrust, which requires no moving mechanical parts. In EHD, corona discharge generates a flow of ions in an electric field between two electrodes; the high-velocity ions transfer their kinetic energy to neutral air molecules through collisions, accelerating the gas and creating thrust. We introduce a fabrication process for EHD thruster based on 355 nm laser micromachining, which potentially allows for greater materials selection, such as fiber-based composites, than is possible with semiconductor-based lithographic processing. Our four-thruster device measures 1.8 × 2.5 cm and is composed of steel emitters and a lightweight carbon fiber mesh. We measured the electrical current and thrust of each thruster of our four-thruster design, showing agreement with the Townsend relation. The peak thrust of our device, at 5.2 kV, was measured to be 3.03 times its 37 mg (363.0 μN) mass using a precision balance. In free flight, we demonstrated liftoff at 4.6 kV.

## Introduction

Insect-scale robotics has been an area of interest for its possible uses in agriculture, search and rescue, and biomedicine, among other areas. The small size and reduced manufacturing cost of insect robots have facilitated micro-robotic research. To date, the primary emphasis in insect-scale robotics has been on drawing inspiration from biology, because biology has found solutions whose existence proves they work. One example is flapping-wing robotic insects [1]. Robots of this type have subsequently incorporated onboard sensors for flight stabilization [2], and lifted off for the first time without needing a wire tether reaching to the ground using a laser power source [3], and subsequently, incandescent light [4]. Other developments include using explosives to break the surface tension of water [5] and RoboFly, which is capable of performing multi-modal locomotion including walking [6], flying, and air-water interfacial locomotion [7]. While flapping-wings are well suited to insect-sized aerial vehicles, they impose a significant cost in terms of mechanical complexity [8]. In this paper, we focus on an alternative means of generating thrust that is not seen in biology: electrohydrodynamic (EHD) thrust. EHD thrust requires sustained high voltage, which may be why it is not observed in biology. From an engineering perspective, EHD has the appealing characteristic that it requires no moving mechanical parts, simplifying fabrication. A recent advance indicating the promise of EHD thrust was fully EHD-powered 2.5 kg airplane [9].

The work in [10] reported the demonstration of a very small EHD-based aircraft, the “Ionocraft” measuring only 3 cm across and consisting of four-thrusters. It was able to take off using an external power source. The strength of that work was how the low outflow velocity from low-voltage EHD thrusters was a good match to the low mass of that device. Additionally, EHD is potentially simpler than flapping-wing flight because even a four-thruster device consists of only a single moving part. However, the device in [10] was fabricated using expensive semiconductor-based cleanroom fabrication facilities. Significant engineering development is required before a small EHD-powered robot can perform aerial locomotion fully autonomously, rendering it capable of useful application. More rapid and less expensive methods to build robots with EHD propulsion could facilitate faster design iteration times, which is highly desired when technology is still in its infancy.

**Fig 1:**
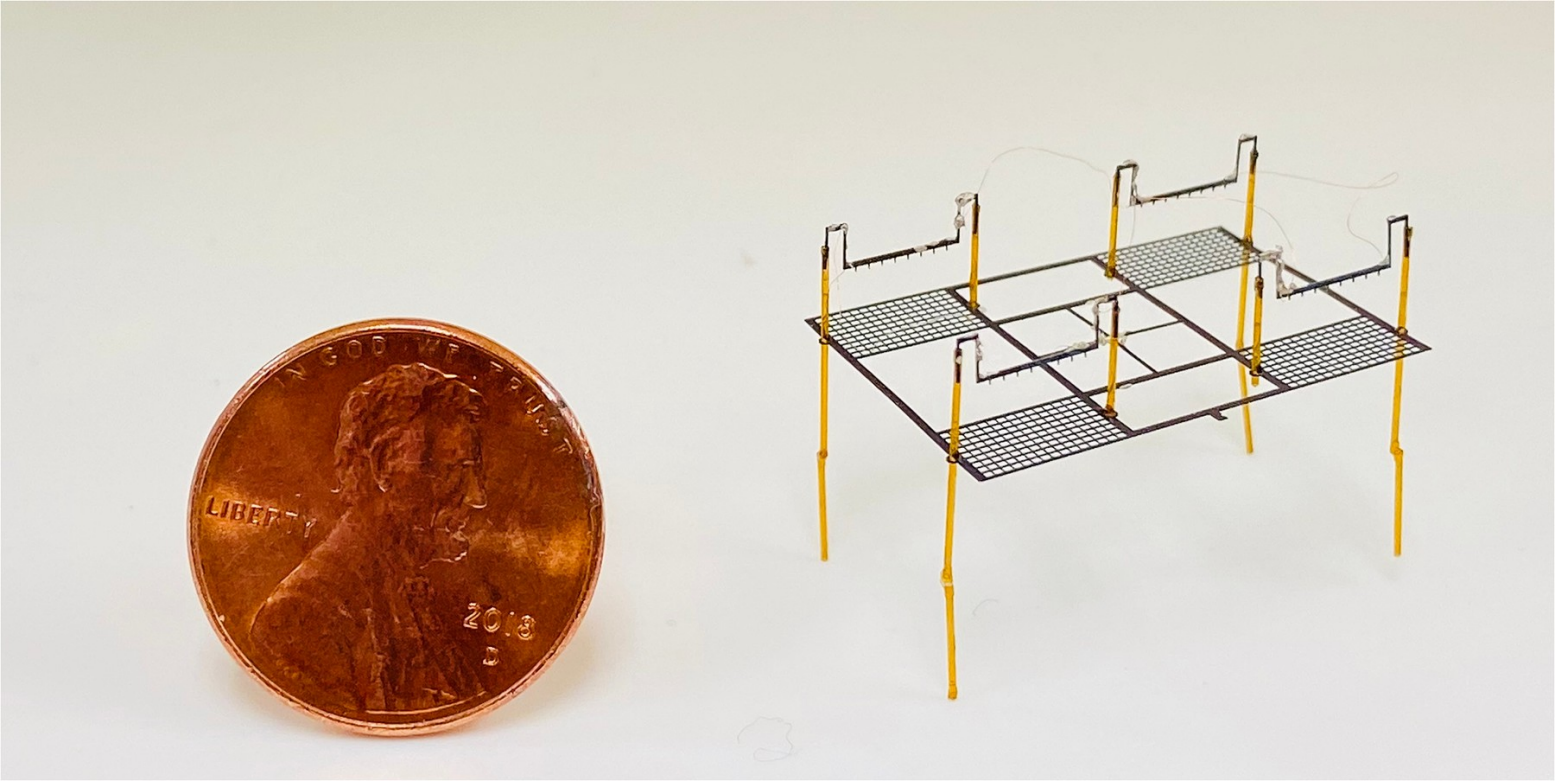
An assembled Quad-thruster robot next to a U.S.penny The 1.8 × 2.5 cm quad-thruster having a mass of 37 mg is shown. The robot components consist of a carbon fiber collector grid, four blue tempered steel emitters, and eight fiber optic glass tubes. All components are hand-assembled using external jigs.

In this paper, we utilize laser machining fabrication to build EHD thrusters for centimeter-sized aerial robots. The process allows for use of a greater variety of electrode materials and eliminates the need for a cleanroom facility. For example, it allows fabricating a complete four-thruster device in a matter of minutes. The quad-thruster device presented in this paper, as shown in Fig 1 has generated thrust exceeding its weight. We report the fabrication methodology and the experimental measurements of the corona current, thrust, and energy transfer efficiency for each individual thruster of the quad-thruster. Finally, we present the takeoff of our quad-thruster.

## Electrohydrodynamics (EHD)

Electrohydrodynamics (EHD) is an interdisciplinary field describing the interaction of fluids with an electric field. Insights into complex multi-physics interactions are essential for understanding EHD flows: (1) ion generation; (2) the ion motion in the electric field; (3) the interaction between the motion of ions and the neutral molecules; and (4) the inertial and viscous forces in the complex flow.

### Corona discharge driven flow

Corona discharge generates a flow of ions in a strong electric field between two electrodes; the high-velocity ions transfer their kinetic energy to the neutral air molecules by collisions that accelerate the gas in the direction of ion drift. This electrohydrodynamic (EHD) flow propulsion phenomenon, also referred to in the literature as ionic wind, is used in many practical applications, such as convective cooling [11, 12], electrostatic precipitators (ESP) [13], plasma-assisted combustion [14], airflow control [15, 16], and as a turbulent boundary layer actuators [17]. The corona induced EHD flow converts electric energy into kinetic energy directly and requires no moving parts. The voltage-current relation during the corona discharge characterizes the ion motion between the electrodes. This phenomenon has been studied since the early 20th century. The classic relationship was derived by Townsend [18] in 1914 and validated for a coaxial corona configuration. Some recent studies modify Townsend’s quadratic relationship to better describe the relationship for different electrode configurations [19–22]. A generalized analytical model for voltage to current and voltage to velocity for EHD driven flow has been recently described [22]; the analytical model has a good agreement with the experimental data in the accelerating flow regions (EHD dominated flow). The analytical study has been extended to determine the voltage-thrust relationship in planar coordinates with and without considering viscous losses and has good agreement with experiments [23] and previous work [24, 25]. Previous studies have reported that maximum velocity for point-to-ring electrode configuration was recorded at 9 m/s [26] and have assessed the use of ionic winds in propulsion applications [24].

Stuetzer [27] presented the first experimental and theoretical analysis of pressure drag produced by the ions, where he determined the pressure generation over a wide range of carrier media. Previous work performed by Masuyama [25] determined the achievable thrust to power ratios of EHD propulsion on the orders of 5-10 N.kW^−1^. Thrust to power ratio was found to be dependent on electrode distance and the potential difference between the electrodes. Similar results were observed for an ionocraft with a wireless power supply onboard and transmitted power up to 100 W to ionocraft at the voltages up to 12 kV [28]. The EHD propulsion can be utilized for UAV propulsion; the experimentally measured maximum thrust density of 15 N.m^−3^ was reported recently [29]. Drew et al. showed that higher thrust density can be achieved for insect-scale robots [10, 30] and EHD flow can be used for flight control.

### Electrohydrodynamic force

A one dimensional model for an EHD thruster yields an expression in terms of the current, distance between the anode and cathode. Space charge effect is ignored here, however, it can be important at high electric field strengths. The current is determined by integrating charge density

$$I=\int J.dA=\int \rho_{e}E\mu dA$$ (1)

where *ρ*_*e*_ is the charge density, *μ* is the ion mobility in the air, *E* is the electric field. An ion mobility value of *μ* = 2 × 10^−4^ m^2^/V-s is assumed in this study. For energy transfer efficiency analysis, consider that thrust is equal to the Coulomb force acting on the volume of fluid between the anode and cathode

$$F=\int \rho_{e}EdV=\frac{Id}{\mu }$$ (2)

where *F* is the thrust, *I* is the ion current, *d* is the distance between electrodes. The corona power can be written as in Eq 3, and efficiency which is defined as *F*/*P* is given by equation Eq 4.

P=IV (3)

$$\frac{F}{P}=\frac{d}{\mu V}=\frac{1}{E\mu }$$ (4)

where *E* is the electric field strength, and *V* is the applied voltage. Drew et al. report the minimum efficiency in their design should be 2 mN/W. The analysis sheds insight into the importance of the electrode distance and applied voltage. Eq 4 shows that for the larger electrode spacing higher efficiency values can be reached as observed by Guan et al. [26]. Related to electrode configuration, it is important to revisit Townsend’s relations [31]

$$I=CV(V-V_{crit})$$ (5)

where *V*_*crit*_ is the onset voltage and *V* is the voltage applied. *C* is a constant related to the geometry of the electrodes [25]. Thrust can be determined using Townsend’s relation

$$F=\frac{CV(V-V_{crit})d}{\mu }$$ (6)

In a practical thruster design, to achieve maximum thrust, the constant *C* needs to be maximized and *V*_*crit*_ needs to be minimized. Other considerations include the effects of non-linear ionization region, secondary flow structures, cathode blockage ratio, the transition from glow to streaming corona discharge and eventually to sparkover. The full optimization of the EHD thruster is beyond the scope of this paper.

## Fabrication

Our fabrication process emphasizes speed and simplicity by minimizing the number of components and fabrication time.

Previous work has used a silicon-on-insulator process for the fabrication of EHD thruster [30]. The emitter and collector electrodes were made from silicon patterned with a photolithographic mask. After a deep reactive ion etch (DRIE) process to ablate through the wafer, the electrodes are once more etched with hydrofluoric acid (HF). In that work, insulating standoffs to separate the emitter from the collector were made from fused silica capillary tubing with an outer diameter of 400 μm. Connections between the tubing and electrodes were made with UV-curable epoxy. Power connections are made with the application of silver epoxy. An external jig was used to align the assembly. device has 13 components (8 capillary tubes, 4 emitters, and 1 collector) in comparison to the most recently reported design for the “Ionocraft” that has 41 components (including sensor components) [10].

**Fig 2:**
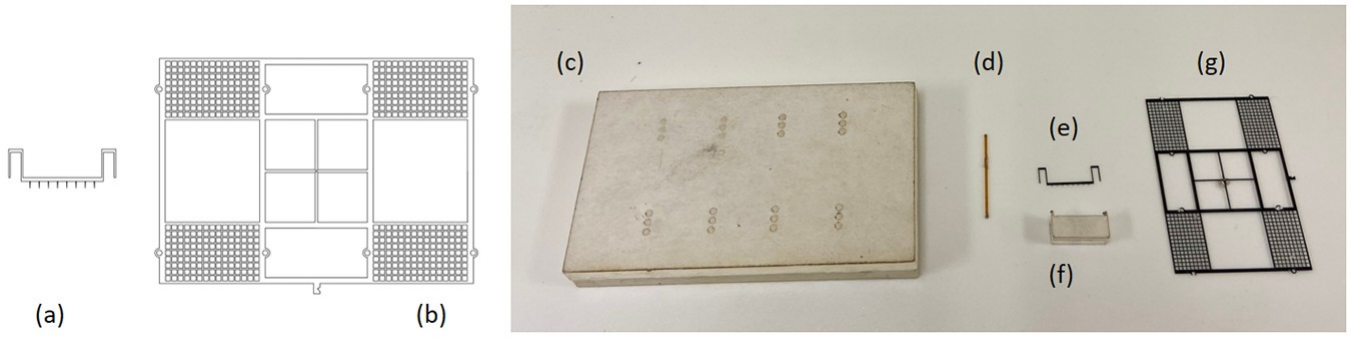
Individual components of the quad-thruster device. Top view of (a) an emitter drawing, (b) a quad-thruster collector grid drawing. (c) A holding jig used for placing eight glass fiber-optic poles shown in (d) and also for keeping the grid in a plane perpendicular to the poles. (d) One of the eight poles required for the assembly. (e) One of the four emitters used in the device. (f) A spacing jig used for keeping emitters at a uniform distance from the grid. (g) A quad-thruster collector grid.

Here, we use laser micro-machining instead of lithographically-patterned silicon. Our laser is a diode-pumped solid-state (DPSS) frequency tripled Nd:Yag laser with 355 nm wavelength (PhotoMachining, Inc., Massachusetts). The DPSS laser output power is 2 W, its beam diameter is 20 μm, and position repeatability is about 3 μm. With this system, we can machine both the emitters and collectors in about ten minutes. Our proposed methodology involves machining using the following steps:

1. Blue tempered steel shim and carbon fiber sheet are laser machined with features for emitters and grid respectively, using the DPSS laser. Their corresponding CAD drawings and actual machined parts are shown in Fig 2(a), 2(b), 2(e) and (2g), respectively.
2. Jig-1 (holding jig) and jig-2 (spacing jig) (Fig 2(c) and 2(f), respectively) are fabricated out of a sheet of acrylic using a standard CO_2_ laser cutter.
3. Poles made out of glass fiber optic tubing of an inner diameter of 250 μm and an outer diameter of 350 μm are used for maintaining a uniform gap between the electrodes. One of the poles is shown in Fig 2(d).

## Design analysis

In this section, we discuss various parameters involved in the design and assembly of the thruster. We walk through design considerations to optimize the thrust generated while selecting values for these parameters.

### Emitter

The emitter (corona electrode) material must be rigid, conductive, and with high curvature features (points). The design analysis of the emitter electrode included material, curvature, number of emitter tips, and orientation.

The material initially used was a 50 μm stainless steel but was changed to a 100 μm blue tempered stainless steel as it was a stiffer and more durable material. We explored different tip angles, starting with 30°, before reducing to 10° and then 5°. A smaller radius of curvature of an emitter tip creates a stronger electric field gradient and higher ion concentration. Due to the limitations on the laser beam diameter used to fabricate the emitter and local heating due to the beam, we found that 5° was the sharpest tip that the machine could fabricate.

The number of emitter tips corresponded to the number of electric field localization for corona discharge to occur. We explored different numbers of tips. In each case, the thruster showed similar thrust values, so we settled on eight tips. The last factor evaluated was the emitter tip orientation. The emitter design in the baseline iteration had electrode tips in the plane parallel to the collector grid. This case involved ions having an initial velocity component in the horizontal direction resulting in loss of kinetic energy that can be avoided by pointing the tips directly towards the grid.

### Collector

The collector is the heaviest of all the components in the thruster. It should have a low blockage ratio to allow the thrust-causing air molecules to flow through it while remaining stiff. We explored different grid spacing and material thicknesses.

We started with a collector grid with 150 μm spacing between grid marks made out of readily available 50 μm stainless steel. Due to issues with weight and bending of the stainless steel grid from the strain of other components, we switched to unidirectional carbon fiber reinforced composite. The carbon fiber sheet was made by laying up the 69 GSM (69 g/m^2^) carbon fiber (TenCate M49J) in 0-90-0 directions. After curing, this lay-up measures about 180 μm thick. The mass of the single thruster carbon fiber grid with this configuration was 5.9 mg, compared to the previous 8.3 mg stainless steel grid. We further optimized spacing and weight by reducing the spacing from 150 μm to 100 μm. We were unable to achieve a functional collector using thinner, 90 μm carbon fiber and reduced grid spacing of 50 μm because of excessive breakage during fabrication (when laser cutter has a spot radius of 20 μm which ends up removing material unevenly on both sides of a grid line). There are 15×9 square openings in the collector grid each with a side length of 100 μm. This works out to a total flow area of 21.6 mm^2^ corresponding to a blockage of 32.75%.

### Quad-thruster design

**Fig 3:**
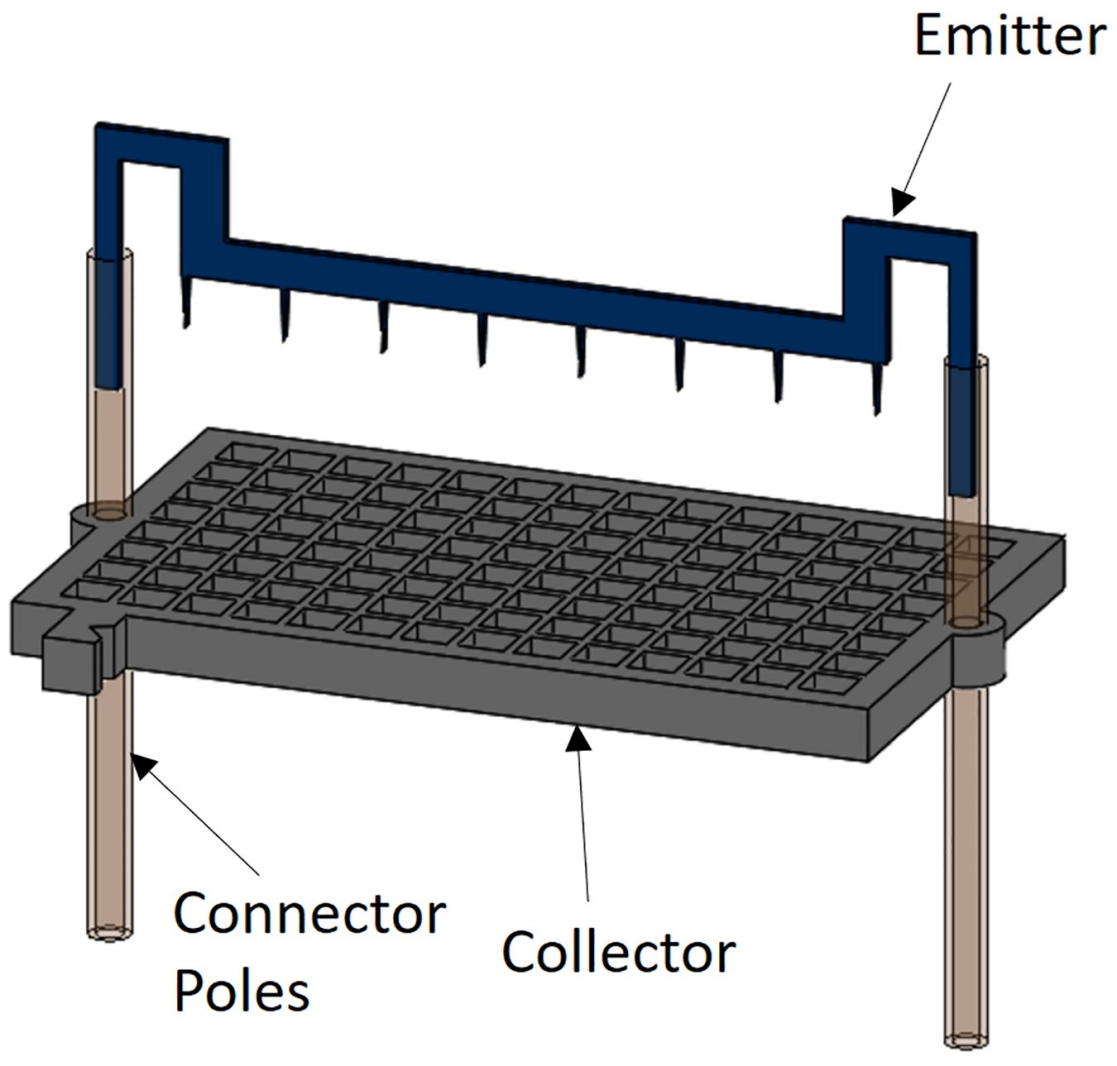
Dimetric view of collector grid, emitter electrode, and connector poles for the current iteration of a single thruster.

After individual thrusters were designed, the next design steps involved putting four of the single thrusters together to make a quad-thruster. In the quad thruster, each single thruster was separated by 7 mm and the inter-electrode spacing is 3.5 mm. Inter-electrode spacing is chosen iteratively based on the observed thrust-to-weight ratio for the current thruster design. Effect of inter-thruster spacing (7 mm) and orientation is neither explored nor optimized in this work. We also created a single thruster, as shown in Fig 3, for testing and performance characterization.

## Assembly

In this section, we discuss the assembly process for the quad-thruster device. The assembly takes about 15 minutes to complete after the components are fabricated. The steps involved in the assembly process are summarized below.

**Fig 4:**
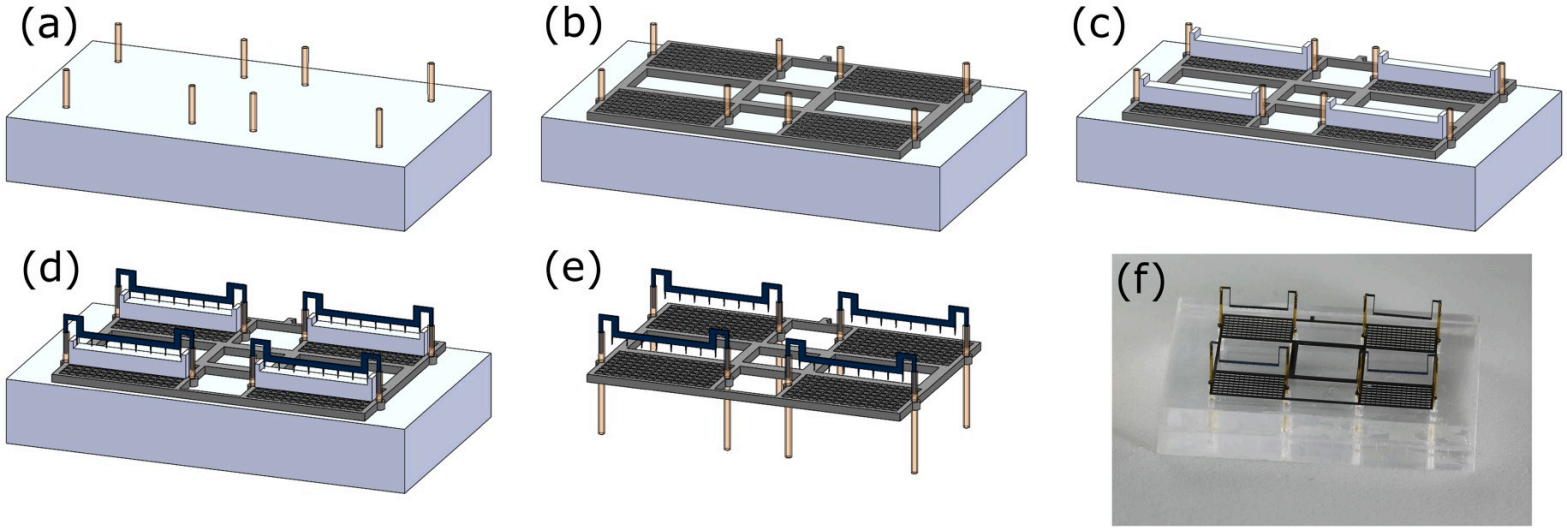
Assembly steps of the quad-thruster device. (a) Fiber-optic glass poles placed into the holes of the holding jig. (b) Grid is then placed on holding jig through the poles. Note: grid thickness exaggerated for 3D view. (c) Four spacing jigs placed on each single collector grid. (d) Four emitters are slid into the poles on top of spacing jigs. (e) Dimetric view of a quad-thruster after the jigs are removed. (f) Picture of a quad-thruster fully assembled in the external jig-1 that is used for assembly.

1. A set of eight poles are placed through holes on jig-1 as shown in Fig 4(a).
2. The grid is carefully aligned and slid through the poles; it is then glued down with these poles from top to avoid accidentally gluing the components with the jig. It then looks as shown in Fig 4(b).
3. Subsequently, four spacing jigs (having a height of 3.5 mm) are placed on each collector grid as shown in Fig 4(c).
4. Four emitters are now slid into the poles on top of each collector grid as shown in Fig 4(d). It is made sure that all of the tips are in contact with their spacing jigs. These emitters are then glued down (Cyanoacrylate) with the poles.
5. Once the glue is dry, all four spacing jigs are removed, and the whole assembly is then taken out of the holding jig. The assembly looks like the CAD shown in Fig 4(e). Fig 4(f) shows a picture of an actual assembly sitting on a holding jig.
6. The whole system is powered through external tethers. The quad-thruster has 2 external wires; a 58-gauge copper wire is attached to one of the inner ends of the emitters such that the connection is closer to the center of the collector grid. The other 58-gauge connection is made to the center of the collector grid. Silver paste is used to make the connections with the corresponding electrode. After an electrical connection is established, a bit of glue is added to further reinforce the joint.

## Experimental method and results

**Fig 5:**
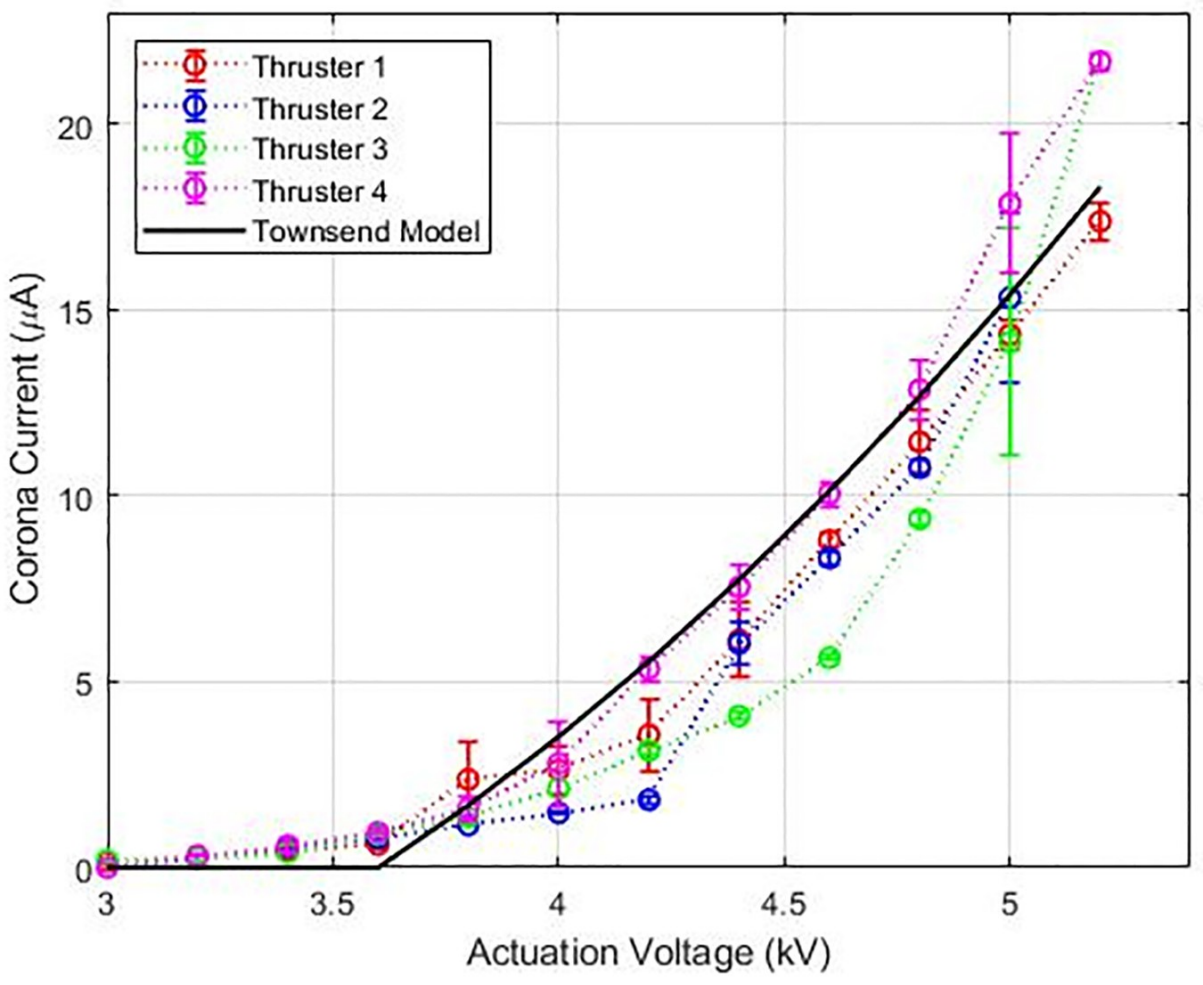
Corona current vs the applied voltage for each individual thruster. The measurements are fitted with a Townsend current model and accurately capture the theoretical trend. Calculated corona onset voltage is 3600 V with a standard deviation of 100 V.

The electrical characterization of each thruster in the quad thruster device was performed first. A high voltage positive DC power supply (Bertan 205B-20R) was used to create the potential difference between the emitter and the collector. The current associated with the discharge was determined from the power supply’s built-in ammeter. Four measurements are taken for the corona current and the generated thrust at each actuation voltage. The sample mean and standard deviation are computed. The error bars used in Figs 5 and 7 denote one standard deviation from the sample mean. Results are shown in Fig 5 for each of the four thrusters that comprise a single unit. The current and voltage trends are similar to previously reported quadratic trends [18, 22, 31]. We’ve fitted our data with the Townsend’s current model shown in Eq (5) using a *C* value of 2.2 and *V*_*crit*_ value of 3.6 kV, and the current-voltage relationship is plotted, showing that our data agree with the model. A peak force of 1.2 mN for the quad thruster is predicted from Eq (6), that corresponds to a theoretical thrust-to-weight ratio of 3.5.

**Fig 6:**
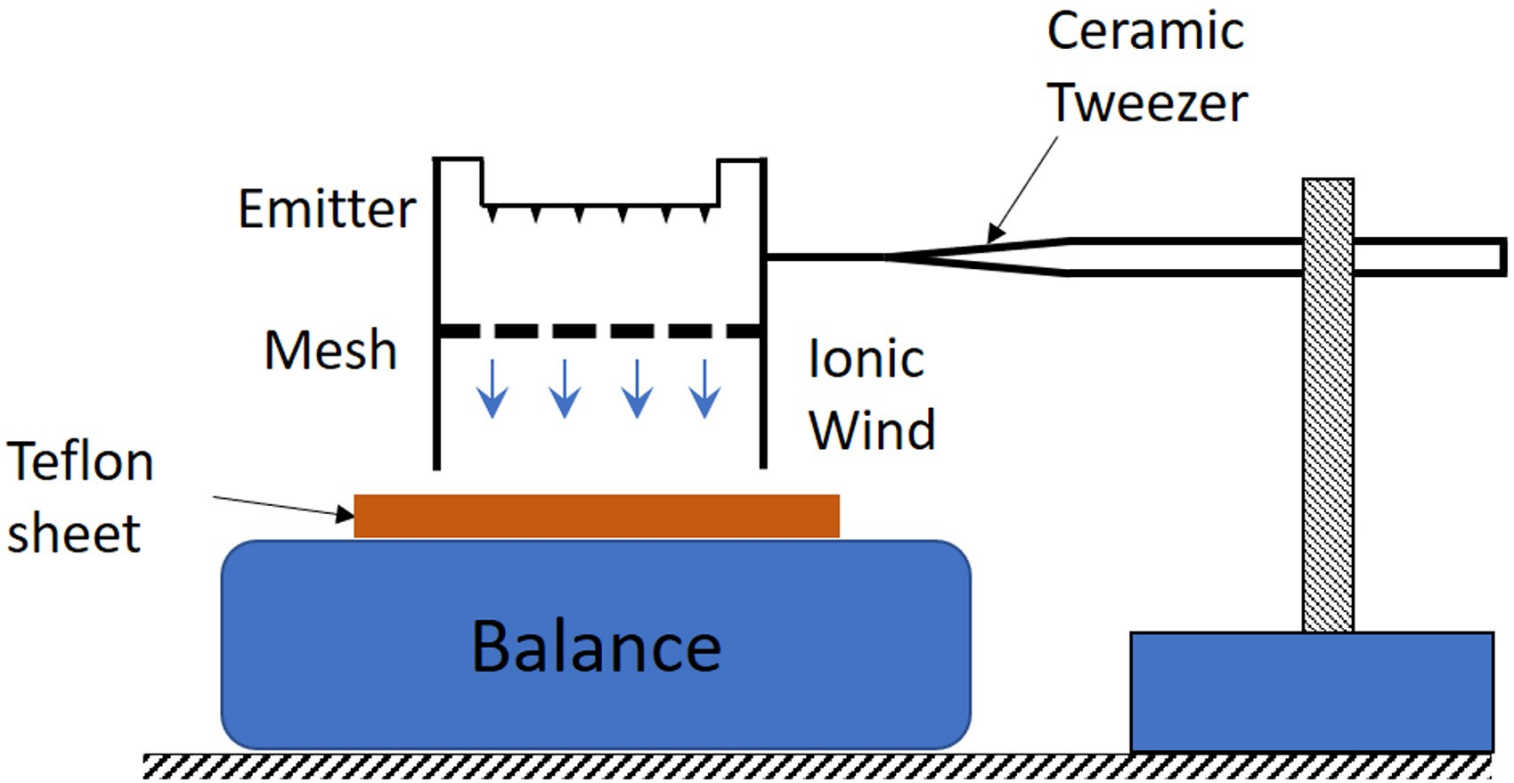
Schematic of thrust measurement apparatus. Thrust generated by the EHD thruster was measured by measuring the force produced by the ionic wind on the precision scale. Tethers are not shown for simplicity and the thruster is not resting on the scale.

**Fig 7:**
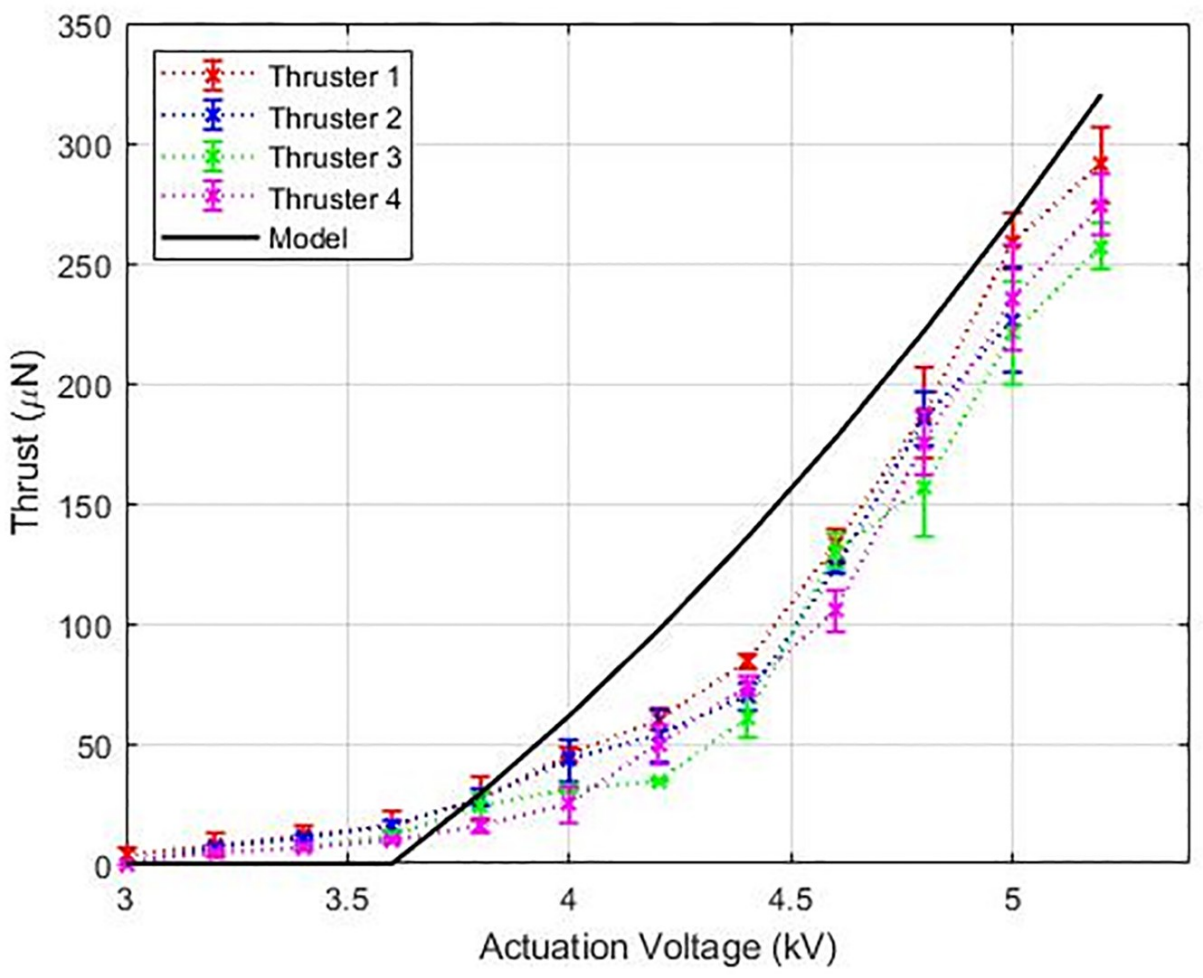
Thrust variation with applied voltage is depicted for each thruster of the quad-thruster device. The input voltages range from 3 kV to 5.2 kV. The Townsend current model shown in Fig 6 is used to compute theoretical upper limit for the measured Coulomb force and the data captures this trend.

The thrust force calculated from the model is validated using experimental thrust measurement as shown in Fig 6; A similar setup has been used in previous studies [24]. The thruster was held directly above the balance with 0.1 mg resolution (Mettler Toledo) such that the collector grid was aligned parallel to the scale surface using a ceramic tweezer. This arrangement, with the thruster fixed, reduces the confounding effect of electrostatic forces acting on the aircraft through the tether wire. The distance between the collector grid and the weight scale is 21 mm. The scale reading was set to zero and each thruster was energized to measure the thrust. A piece of Teflon was placed between the balance plateau and the collector to electrically isolate the balance and to avoid any leakage current. The measured thrust is the force exerted by the accelerated ionic wind on the precision scale. It can be seen that the thrust increases with the voltage applied across the electrodes as shown in Fig 7. The thrust trends follow the previously reported quadratic relationship with the applied voltage [23–25]. The maximum thrust generated occurs immediately before the sparkover is initiated, at which point thrust drops to zero and destroying the mesh. The peak force generated by each thruster was around 260 μN with a standard deviation of 20 μN. The model predictions were higher than the experimental results, but the results captured the trend. The difference between the model and experiments can be attributed to blockage and drag losses as shown in [23, 24]. The model described in Eq (6) helped us to validate the thrust measurements and can form the basis for future constrained parametric optimization studies of the quad-thruster device.

**Fig 8:**
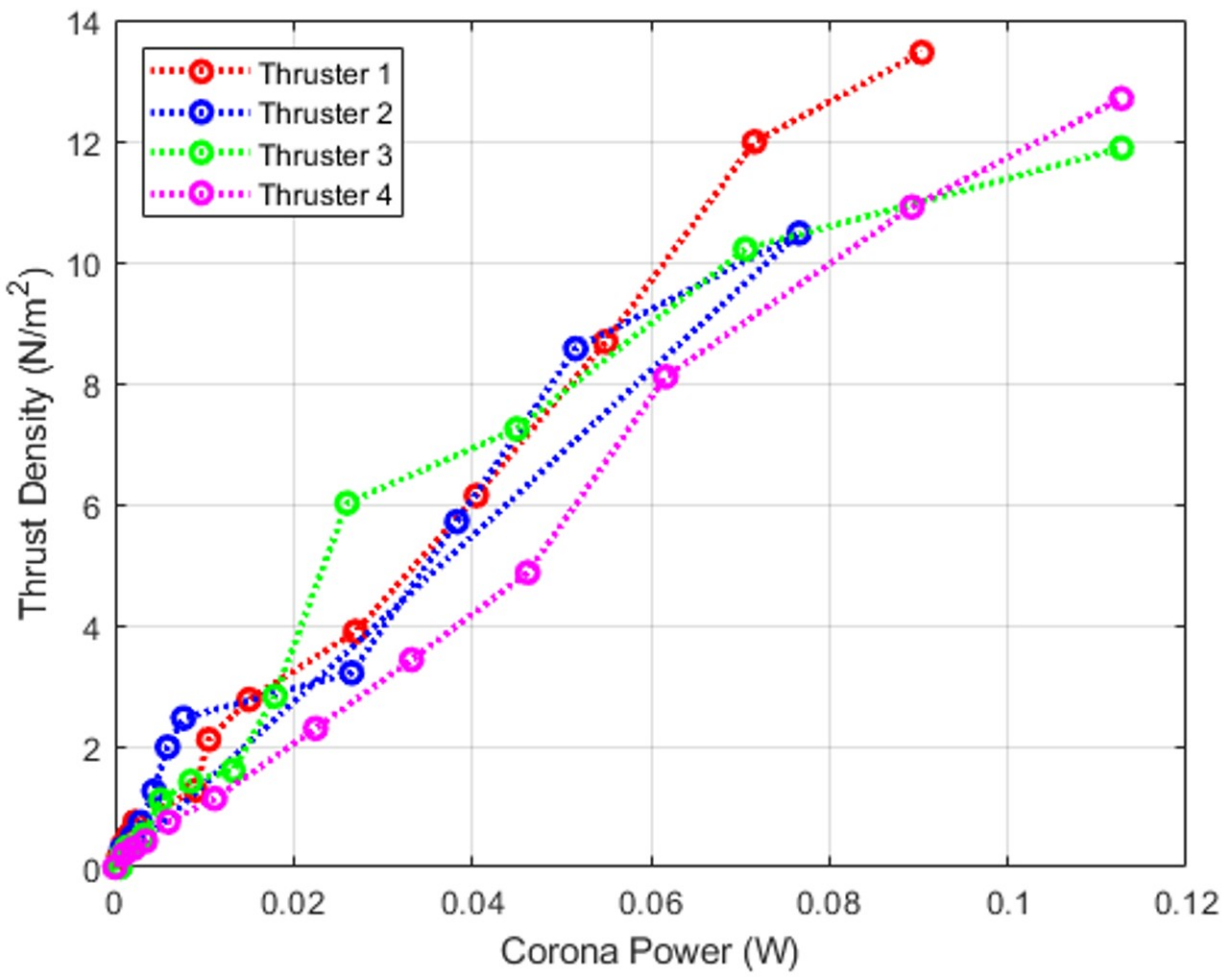
Efficiency in terms of thrust density versus the corona power for each thruster in the quad-thruster device. The data points shown displayed are mean values of thrust density and corona power.

Thrust density and efficiency are important parameters in understanding the working and performance of EHD thruster compared to other designs. Thrust density is defined as the amount of thrust generated per unit area, whereas efficiency is defined as thrust per unit power. Thrust density for EHD thrusters is calculated from the effective area where EHD flow exists, i.e., the mesh area. Fig 8 shows how the thrust density varies with the corona input power of EHD thruster. The electrical power was calculated from Eq 3. A maximum thrust of 0.295 mN corresponds to 13.67 N/m^2^ thrust density achieved at an input electrical power (aka corona power) of 90.4 mW. Therefore, the thrust density per unit power for the EHD thruster is 151.17 N/m^2^W. The efficiency is about 3.265 mN/W. This data can be compared to a piezo-actuated flapping-wing such as the RoboFly [6], which has a measured efficiency of 12.2 mN/W. For a thrust of 0.736 mN, an input power of 60 mW, and a 308 mm^2^ effective swept area of the wing, the thrust density is 2.39 N/m^2^. Therefore, the thrust density per unit power is 39.8 N/m^2^W. Therefore, while the efficiency of the EHD thruster is lower than a flapping-wing robot of comparable size (the efficiency of the flapping-wing robot is 3.74 times higher), the thrust density per unit power consumed is 3.8 times higher for the EHD thruster. This is important because the thrust density correlates to the mass of the thruster, and therefore this metric represents a scale-independent (and propulsion-type-independent) measure of efficiency.

**Fig 9:**
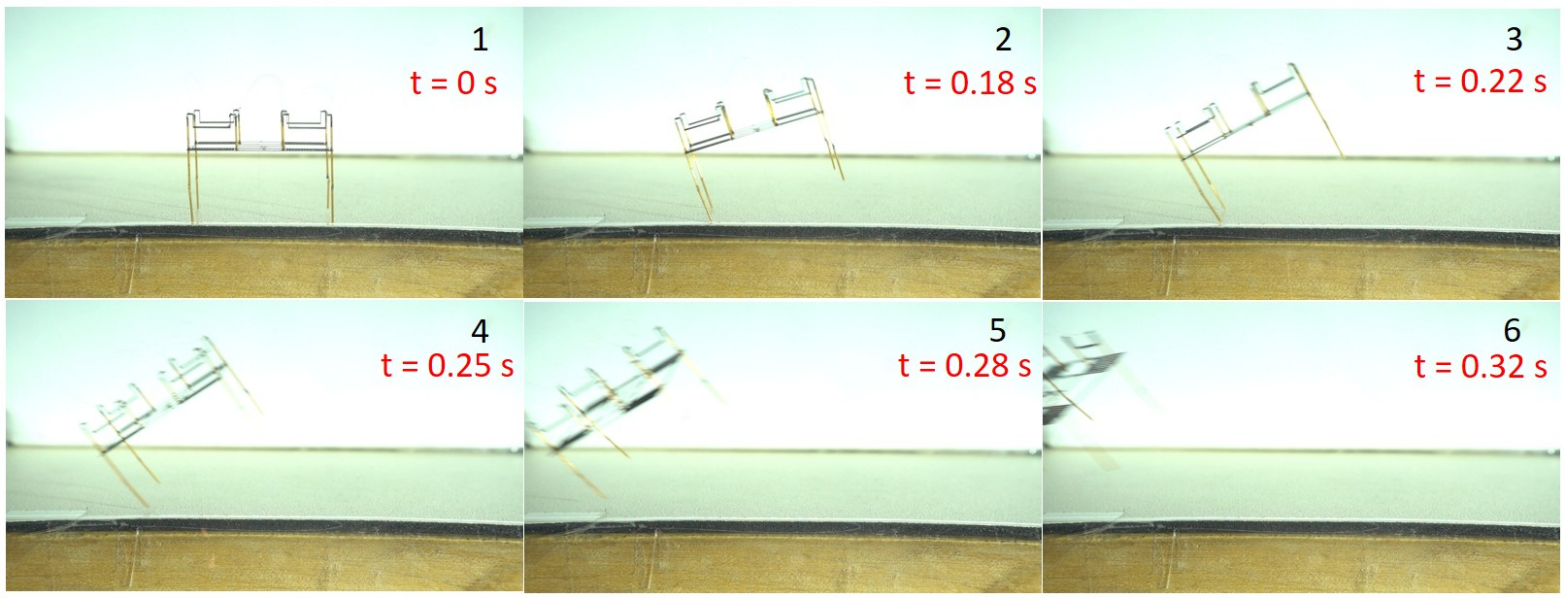
Frames captured at a frame rate of 240 fps from the quad-thruster in flight. The thruster is resting on the table with the collector connection dangling down and the emitter connection straight up.

For free flight experiments, the device was placed on a wooden table. The four emitters are interconnected with 51 gauge wires as mentioned above in the assembly section and the quad thruster was actuated using two 58 gauge copper wires in front of a high-speed camera (Sony RX100). One 58-gauge connection is attached from the top to one of the inner ends of an emitter and the other connection is attached to the center of the collector grid. The power tethers were held using ceramic tweezers and strain relieved. The inner legs were removed and attached to the outer legs of the thruster to increase the height and diminish the electrostatic interaction with the takeoff plane. With a voltage of 4.6 kV, lift off of quad thruster was achieved. Fig 9 provides an image sequence from the flight for the first 0.32 seconds before the wires touched each other, which ended the free flight. This conclusively shows that the device is able to lift its own weight and in the future, a vertical liftoff can be achieved by trimming the thruster as illustrated in [32].

Finally, we conclude this section by comparing the performance of the single-emitter thrusters of our four-thruster device to the single-emitter, the four-thruster device presented in [30] (which did not directly measure thrust measurements). Hence, to compare the performance of the two arrays, the thrust generated (estimated from the takeoff video presented in [30]) and thrust-to-weight ratio are chosen as the criteria. The single emitter version of the Ionocraft takes off at 2400 V with a corona current measuring close to 20 μA. This works out to a corona power of 0.048 W, and from the observed peak acceleration of the Ionocraft, they conclude that the corresponding thrust is approximately 200 μN which amounts to a thrust-to-weight ratio of 2.04. At the same input power, our quad-thruster device generates 675 μN which gives a thrust-to-weight ratio of 1.86. Table 1 includes a comprehensive summary of manufacturing methodology and performance comparison between the Ionocraft and our laser-fabricated quad-thruster device.

**Table 1:** Comparison of our work with earlier work by Drew [30].

Comparison Criterion	Drew [30]	Our work
Electrode Material	Silicon	Carbon fiber (grid) Stainless steel (emitter)
Total weight (μN)	98 μN (10 mg)	362.6 μ N (37 mg)
Thrust at 0.048 W (μN)	200 μN	675 μN
thrust-to-weight ratio at 0.048 W	2.04	1.86
Assembly time in minutes	30	15
Clean-room facility	Required	Not required

## Conclusion

A quad thruster was fabricated using UV laser micro-machining and the performance of the quad thruster is characterized. Measured current and thrust values are in good agreement with the EHD theory. The variation across all the four thrusters in the device is minimal and the lift-off of the quad-thruster device is demonstrated. The device was able to lift its weight, as indicated by thrust measurements and free-flight connected to a wire tether. The thrust-to-weight ratio of our device at takeoff voltage of 4.6 kV is 1.38 with a power consumption of 0.037 W. The peak thrust-to-weight ratio of our thruster at the maximum actuation voltage of 5.2kV (with a typical operating range of 3.6kV to 5.2kV) is 3.03 with a power consumption of 0.104 W, which is slightly below the ratio of 4.5 reported previously [33]. We used fewer emitter rows than [33], and we expect that adding more rows, will substantially increase lift with little added weight. We also believe there is ample opportunity to reduce the mass of our device through the use of thinner and lighter material. We plan to conduct an exploration of different designs in finite element simulation to explore the configuration space in greater detail. All of this work will contribute significantly to boosting the thrust-to-weight ratio of our quad-thruster device which is fundamental for EHD propelled centimeter-scale aerial robots.

The fabrication time, from raw materials to a complete quad-thruster device takes about 25 minutes and this approach allows for great flexibility in the selection of materials. The process is viable and a faster alternative to a silicon-on-insulator fabrication process of an EHD thruster at the laboratory prototyping stage. This compares favorably with the process reported in literature [30], which takes 2-3 days at best. In addition to short fabrication time, we remark that laser micro-fabrication allows for a much more diverse material set. While silicon has a high strength-to-weight ratio, other materials can provide better performance for certain applications, such as the even higher strength-to-weight ratio of unidirectional carbon fiber composites. Furthermore, if other materials may improve the lifetime of the sharp emitter tips, it is almost certainly possible to machine it using a DPSS laser, and the fabrication of these electrodes does not require a cleanroom facility.

The path to an autonomy mandates advancement on several fronts with various components as payload. A small on-board camera (as demonstrated in [34] for a flapping-wing insect-scale robot) along with other sensing units such as IMUs (has mass of 37 mg in [10]) can be added, which will be instrumental in controlled flight and sensing around surroundings. Future work also includes implementing on-board power supply and associated power-electronics. Until recently, micro-robots have been powered through external connections. The Autonomous Insect Robotics Lab at the University of Washington has developed light weight circuit that requires no battery and provides wireless power to a robot [3]. This circuit was capable of developing 200 V to drive piezo actuators in a 100 mg package. We expect that a similar approach could extend to the kV potential differences needed for EHD thrusters.

## Appendix

### Future flight control

**Fig 10:**
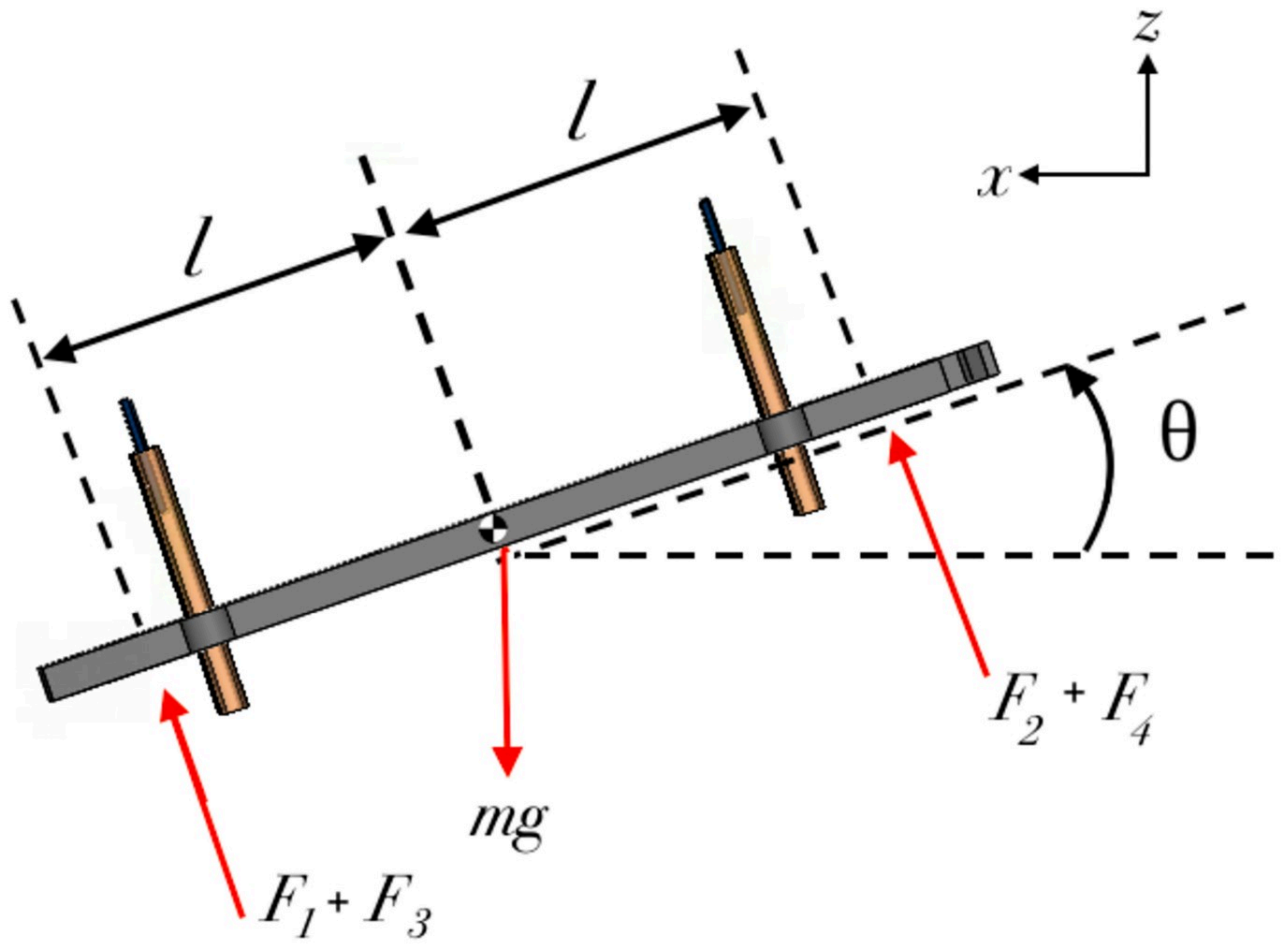
Free-body diagram of a quad-thruster in a side view to demonstrate the pitch/roll control. The pitch/roll control of a quad thruster is similar to multicopters. Thrust generated by i-th thruster is denoted by *F*_*i*_. For positive pitch/roll angle, *θ*. *F*_1_+*F*_3_ and *F*_2_+*F*_4_ can be controlled actively to stabilize the thruster at a desired orientation *θ*.

The goal of using four individual EHD thrusters is to allow for pitch and roll control similar to that of a quadcopter. We anticipate the controller for the quad-thruster will be similar to that of the four wings insect scale flapping-wing robot developed in [35], and quadcopters in general. The free-body diagram for the pitch/roll dynamics is shown in Fig 10. The thruster is symmetric about its roll and pitch axes which results in an identical formulation for the associated dynamics. Yaw control is left for future work. The Pitch/Roll dynamics are described as follows.

$$\begin{array}{cc} I_{p}\ddot{\theta } & =[\left(F_{2}+F_{4}\right)-\left(F_{1}+F_{3}\right)]l\\ m\ddot{z} & =[\left(F_{2}+F_{4}\right)+\left(F_{1}+F_{3}\right)]\text{cos}\theta -mg\\ m\ddot{x} & =[\left(F_{2}+F_{4}\right)+\left(F_{1}+F_{3}\right)]\text{sin}\theta \end{array}$$ (7)

The above set of Eq (7) are derived from Fig 10, where *I*_*p*_ is the moment of inertia of the thruster about the pitch/roll axis, $\ddot{\theta }$ is the angular acceleration, m is the mass of the thruster, and *F*_*i*_ is the thrust force generated by i-th thruster. This shows that by modulating thrust forces *F*_*i*_, the thruster is fully controllable in the *x* − *z* plane and due to the aforementioned symmetry, it is also fully controllable in the *y* − *z* plane.

## Supporting information

S1 File (BIB)
