# Correction: A laser-microfabricated electrohydrodynamic thruster for centimeter-scale aerial robots

**DOI:** 10.1371/journal.pone.0238267

**Published:** 2020-08-20

**Authors:** Hari Krishna Hari Prasad, Ravi Sankar Vaddi, Yogesh M. Chukewad, Elma Dedic, Igor Novosselov, Sawyer B. Fuller

The third author, Yogesh M. Chukewad, should be noted as contributing equally to this manuscript with the first two authors, Hari Krishna Hari Prasad and Ravi Sankar Vaddi.
